# OH-Radical Oxidation of Lung Surfactant Protein B on Aqueous Surfaces

**DOI:** 10.5702/massspectrometry.S0077

**Published:** 2018-11-21

**Authors:** Shinichi Enami, Agustín J. Colussi

**Affiliations:** 1 National Institute for Environmental Studies; 2 Linde Center for Global Environmental Science, California Institute of Technology

**Keywords:** air pollution, lung surfactant, reactive oxygen species, interfacial reaction, protein

## Abstract

Air pollutants generate reactive oxygen species on lung surfaces. Here we report how hydroxyl radicals (·OH) injected on the surface of water react with SP-B_1–25_, a 25-residue polypeptide surrogate of human lung surfactant protein B. Our experiments consist of intersecting microjets of aqueous SP-B_1–25_ solutions with O_3_/O_2_/H_2_O/N_2_(g) gas streams that are photolyzed into ·OH(g) *in situ* by 266 nm laser nanosecond pulses. Surface-sensitive mass spectrometry enables us to monitor the prompt (<10 μs) and simultaneous formation of primary O*_n_*-containing products/intermediates (*n*≤5) triggered by the reaction of ·OH with interfacial SP-B_1–25_. We found that O-atoms from both O_3_ and ·OH are incorporated into the reactive cysteine Cys_8_ and Cys_11_ and tryptophan Trp_9_ components of the hydrophobic N-terminus of SP-B_1–25_ that lies at the topmost layers of the air–liquid interface. Remarkably, these processes are initiated by ·OH additions rather than by H-atom abstractions from S–H, C–H, or N–H groups. By increasing the hydrophilicity of the N-terminus region of SP-B_1–25_, these transformations will impair its role as a surfactant.

## INTRODUCTION

Human lung alveoli are covered by a 0.1–0.5 μm thick epithelium lining fluid (ELF) that has a large surface area ∼885,000 cm^2^ (*vs.* the ∼4,500 cm^2^ of the airway).^1)^ ELF contains surfactant proteins B and C (∼10 wt% of total ELF surfactant material),^1–6)^ in addition to water-soluble antioxidants such as glutathione ([GSH] ∼100–500 μM), ascorbic acid ([Asc] ∼100 μM),^1,7)^ uric acid, and liposoluble α-tocopherol.^7–11)^ SP-B functions reduce the energy required to expand lungs during aspiration and prevent their collapse upon exhalation.^12)^ A recent simulation study revealed that SP-B induces the formation of bilayer reservoirs by monolayer folding, helps attaching the disconnected bilayer aggregates to a monolayer at the air–water interface, and facilitates lipid transfer between these structures.^13)^ SP-B deficiency is known to cause severe lung dysfunction and inflammation.^14)^ Human infants genetically lacking SP-B cannot survive.^15)^ It is apparent that the oxidative degradation of SP-B’s surface activity should induce acute syndromes.^2,16–19)^

Recent studies suggested that the inhalation of particulate matter (PM) produces ·OH in ELF.^20–22)^ *In vivo* studies have shown that ·OH produced in ELF exposed to O_3_(g) enhances bronchoalveolar permeability.^23)^ Given the abundance of antioxidants in ELF (see above), SP-B oxidative damage can be triggered only by ·OH produced or deposited close to SP-B.^24)^ Because inhaled gas-phase oxidants are very reactive and the SP-B surfactant is present at the ELF surface, *the relevant reactive events are expected to take place at the air–water interface rather than in the bulk liquid*. A previous study found that the heterogeneous reaction of O_3_(g) with SP-B_1–25_ (a model 25-residue polypeptide of human SP-B)^25)^ in water–methanol droplets yields several products that incorporate O-atoms in a few seconds.^3)^ The products from the droplets exposed to O_3_(g) or the solution treated O_3_-bubbling were found to be quite different,^3)^ implying that the mechanism at the gas–liquid interface is different from that in bulk solution.^7)^ The authors also generated reactive intermediates in bulk water with Fenton’s reagent (Fe^2+^+H_2_O_2_) to test whether the chemistry actually involved ·OH radicals. However, we recently found that the Fenton reaction at the air–water interface produces oxo-ferryl species (Fe^IV^=O) in high yields, which behave as O-atom donors rather than as H-abstracting species.^26–30)^ In fact, a recent study suggested that Fenton’s reaction produces <10% ·OH in bulk water at physiological pH 6–7.^28)^ Since the production of Fe^IV^=O is significantly enhanced in Fenton’s reaction at the air–water interface,^26)^ establishing the mechanism of the oxidation of SP-B_1–25_ initiated by ·OH at the air–water interface requires experiments involving a direct, *bona fide* source of ·OH *in situ*.

In this paper we report a mass-spectrometric study specifically designed to address these issues, in which aqueous SP-B_1–25_ (a 25-residue polypeptide NH_2_-^1^FPIPLPYCWLCRALIKRIQAMIPK^25^G-COOH) reacts with gas-phase ·OH at the air–water interface. SP-B_1–25_ has been used as a representative model peptide that mimics SP-B functions, because of the similarity of their chemical and physical properties.^4,6)^ Our experiments were conducted in a novel setup in which aqueous SP-B_1–25_ microjets were intersected with gas-phase ·OH streams generated in close proximity to the surface *via* pulsed laser photolysis of O_3_(g)/H_2_O(g)/O_2_(g)/N_2_(g) mixtures,^31–36)^ while continuously monitoring the composition of the interfacial layers of the microjets *via* pneumatic ionization mass spectrometry.^37–39)^

## EXPERIMENTAL SECTION

The present experimental setup is essentially the same as the one reported elsewhere.^31)^ The charged product species generated on the surface of SP-B_1–25_(aq) microjets during τ ∼10 μs contact times (τ is the lifetime of the microjets before being pneumatically nebulized) with O_3_(g) or ·OH(g) streams are monitored *in-situ* by online mass spectrometry (Fig. S1, Agilent 6130 Quadrupole LC/MS Electrospray System).^31)^ O(^1^D) generated by 266 nm photons reacts with excess H_2_O(g) ([H_2_O(g)] ∼7.6×10^17^ molecule cm^−3^) to form ·OH radicals within ∼6 ns (from *k*_1_(O(^1^D)+H_2_O)=2.2×10^−10^ cm^3^ molecule^−1^ s^−1^) (see [⋅OH(g)] estimates in SI). These conditions ensure that the detected species correspond to the truly initial stages of reaction.^34–36)^ The all-^12^C isotopomer of SP-B_1–25_≡Phe_1_-Pro_2_-Ile_3_-Pro_4_-Leu_5_-Pro_6_-Tyr_7_-Cys_8_-Trp_9_-Leu_10_-Cys_11_-Arg_12_-Ala_13_-Leu_14_-Ile_15_-Lys_16_-Arg_17_-Ile_18_-Gln_19_-Ala_20_-Met_21_-Ile_22_-Pro_23_-Lys_24_-Gly_25_, has a molecular mass of 2928 Da. Samples are injected at 100 μL min^−1^ into the spraying chamber of the mass spectrometer through a grounded stainless steel needle (100 μm bore) coaxial with a sheath issuing nebulizer N_2_(g) at a high velocity (*v*_g_≈160 m/s).^38)^ The surface specificity of our experiments has been demonstrated previously.^26,37,38,40,41)^ It should be emphasized that the products we observe are formed when gaseous reactants collide with the intact aqueous jets as they emerge from the nozzle, *i.e.*, before jets are broken up into sub-micron charged droplets by the nebulizer gas.^26)^ Since 266 nm pulses flash every 100 ms, and the microjets breaks up within ∼10 μs after being ejected from the nozzle, it is safe to assume that the observed phenomena always take place on the surface of fresh solutions.^31)^

We verified that in the absence of O_3_(g) neither reactant signals are affected nor new product signals appear upon 266 nm pulsed irradiation, thereby excluding the photolysis SP-B_1–25_ (aq) as the source of detected products (see Fig. S2). In our experiments, ⋅OH(g) will stick to the surface of water in nearly every collision,^31,33–35,42–46)^ and react with SP-B_1–25_ (R1), or dimerize into H_2_O_2_ (R2) at the air–water interface. 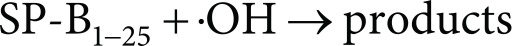
(R1)
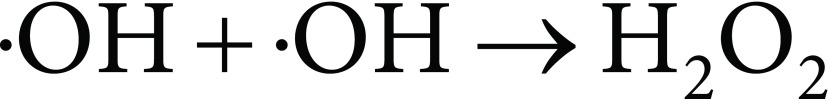
(R2)

The preference of ·OH for interfacial layers relative to the bulk liquid is supported both by theoretical and experimental studies.^31,32,34,43–46)^ This effect should enhance the probability of ·OH reactions with hydrophobic residues at the air–water interface before it diffuses into bulk phase.

## RESULTS AND DISCUSSION

**Figure 1:**
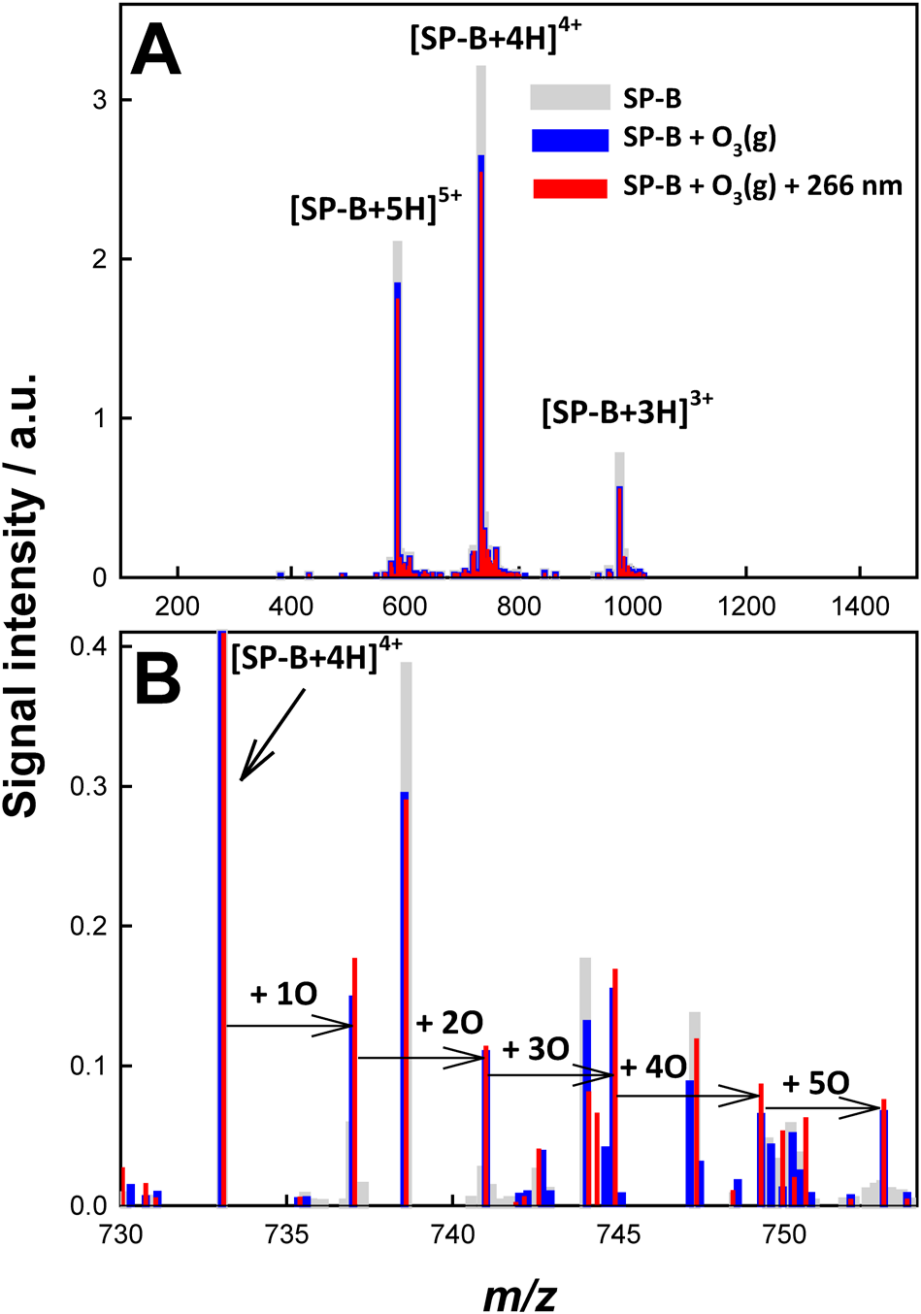
Fig. 1. A) Positive ion mass spectra of aqueous 43 μM SP-B_1–25_ microjets in O_2_(g)/H_2_O(g)/N_2_(g) mixtures (gray), or exposed to ∼500 ppmv O_3_(g) in O_2_(g)/H_2_O(g)/N_2_(g) mixtures in the absence (blue)/in the presence (red) of 40 mJ 266 nm pulses. 1 ppmv=2.46×10^13^ molecules cm^−3^. B) Spectra of [SP-B_1–25_+4 H^+^]^4+^ and its oxidation products in the 730–760 Da range.

Figure 1A shows a positive ion mass spectrum of 43 μM SP-B_1–25_(aq) microjets exposed to O_2_(g)/H_2_O(g)/N_2_(g), and to O_3_(g)/O_2_(g)/H_2_O(g)/N_2_(g) with the 266 nm laser on and off. The three major peaks at *m*/*z*=586.7, 733.0 and 977.0 correspond to multiply protonated [SP-B_1–25_+*m* H]*^m^*^+^ for *m*=5, 4 and 3, respectively. It is apparent that [SP-B_1–25_+4 H]^4+^ [NH_2_-FPIPLPYCWLCR(H^+^)ALIK(H^+^)R(H^+^)IQAMIPK(H^+^)G-COOH)^4+^ is the most abundant species under present conditions. We found that the relative abundances of the most protonated species increase at lower SP-B_1–25_(aq) concentrations (Fig. S3), a phenomenon that we tentatively ascribe to decreased repulsion among sparser polycationic chains in the air–water interfacial layers sampled by our technique. Hereafter, our analysis will largely focus on the evolution of the *m*/*z*^+^=733.0 [SP-B_1–25_+4 H]^4+^ species upon variations of experimental conditions.

All [SP-B_1–25_+*m* H]*^m^*^+^ signals decrease and new products appear (Figs. 1A and 1B, blue trace) in the presence of O_3_(g) without 266 nm. Thus, SP-B_1–25_ is found to be reactive toward O_3_, in accordance with literature reports.^3,6,47)^ In Fig. 1B, the *m*/*z*^+^=737=[2928+4 (+4 H)+16 (+O)]/4 signal is assigned to [SP-B_1–25_+4H+O]^4+^. The *m*/*z*^+^=741=[2928+4 (+4 H)+32 (+2O)]/4 signal is [SP-B_1–25_+4 H+2 O]^4+^, *i.e.*, *m*/*z*^+^=733+4 *n* is [SP-B_1–25_+4 H+*n* O]^4+^ (*n*=1–5). We did not detect *n*≥6 products in our experiments. Importantly, the application of 266 nm laser pulses *enhances* all product signals (Fig. 1B, red trace), thereby suggesting that the detectable products of SP-B_1–25_ oxidation by O_3_ and ⋅OH might be identical. This finding is in accordance with previous reports on the ozonation of amino acids,^48)^ and the ⋅OH-initiated oxidation of proteins.^49)^

**Figure 2:**
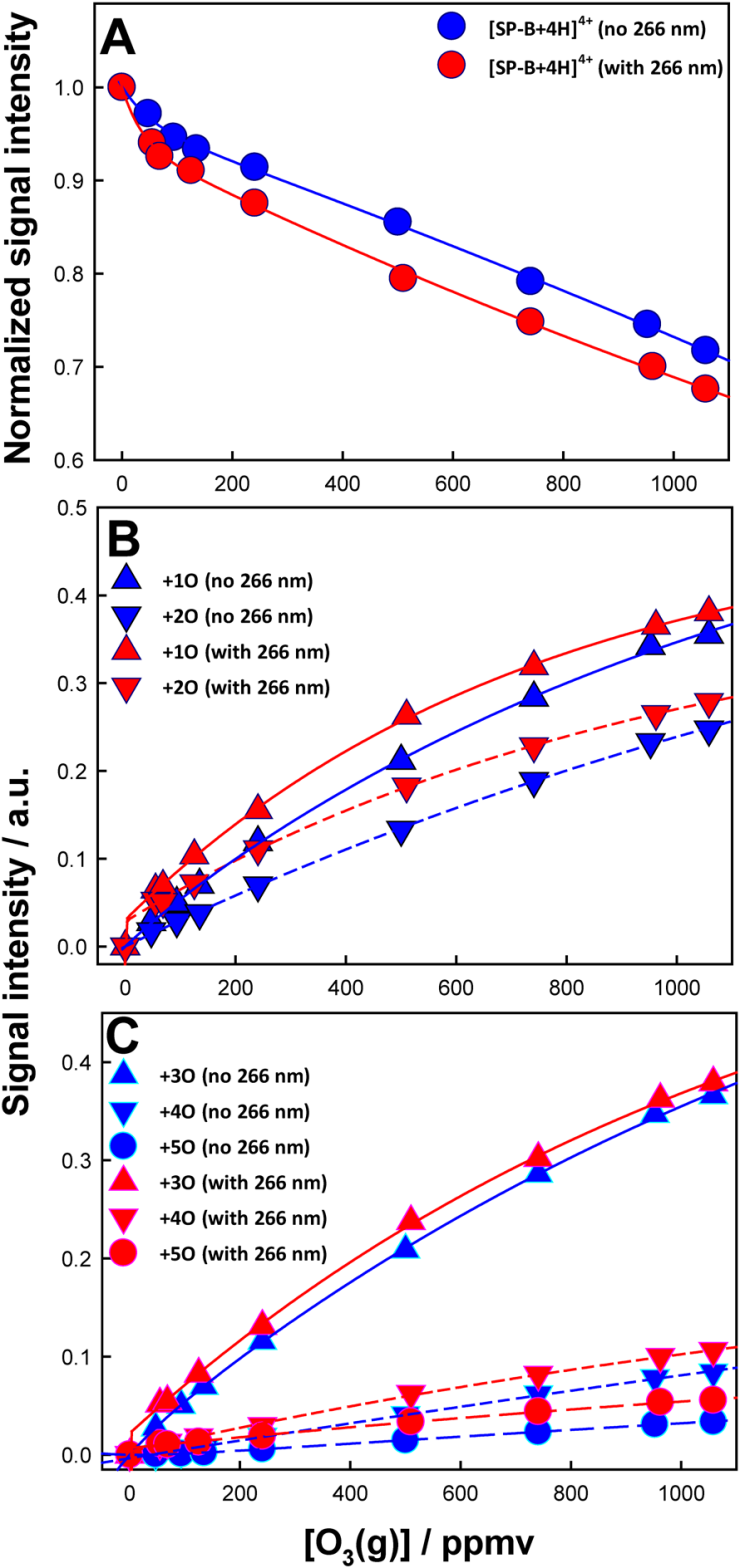
Fig. 2. Reactant (A) and products (B, C) mass spectral signal intensities from aqueous 43 μM SP-B_1–25_ microjets exposed to O_3_(g)/O_2_(g)/H_2_O(g)/N_2_(g) mixtures in the absence (blue) or presence (red) of 266 nm laser beams (40 mJ pulse^−1^) as a function of the O_3_(g) mixing ratio; 1 ppmv=2.46×10^13^ molecules cm^−3^.

Figure 2 shows [SP-B_1–25_+4 H+*n* O]^4+^ mass spectral signal intensities from SP-B_1–25_(aq) microjets exposed to O_3_(g)/O_2_(g)/H_2_O(g)/N_2_(g) mixtures under on and off 266 nm pulses as functions of [O_3_(g)]. It is apparent that the depletion of SP-B_1–25_ is more extensive and [SP-B_1–25_+4 H+*n* O]^4+^ (*n*=1–5) production is enhanced upon irradiation. Note the simultaneous appearance of all *n*=1–5 [SP-B_1–25_+4 H+*n* O]^4+^ products (see below). Figure S4 compares the bi-exponential depletion of [SP-B_1–25_+3 H]^3+^, [SP-B_1–25_+4 H]^4+^ and [SP-B_1–25_+5 H]^5+^ signal intensities under the same conditions of Fig. 2. The fact that the extent of [SP-B_1–25_+3 H]^3+^ depletion is significantly larger than for the other protonated species suggests structural changes that depend on *m*.^50–52)^ Species of smaller *m* values behave as if they increase the exposure of the more hydrophobic (surface-active) amino acid residues to O_3_ and ·OH. This is consistent with experiments showing that more hydrophobic long-chain species react more extensively with ·OH(g) than the less hydrophobic shorter-chain ones.^31,32,34,35)^ This is also consistent with a recent report showing that antioxidant activity of free amino acids in Fenton’s reaction increases with hydrophobicity.^53)^

**Figure 3:**
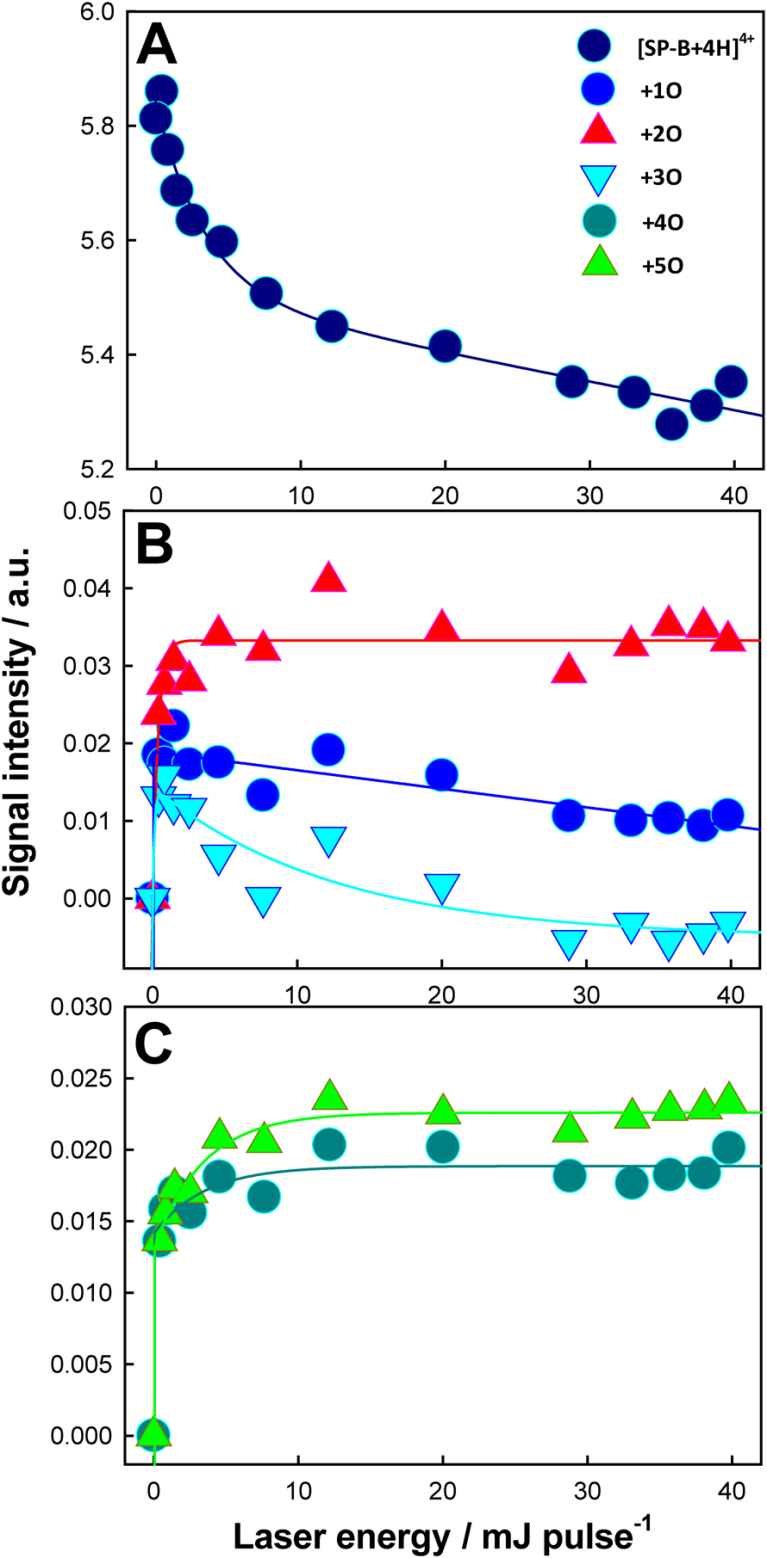
Fig. 3. Reactant (A) and products (B, C) mass spectral signal intensities from aqueous 43 μM SP-B_1–25_ microjets exposed to O_3_(g)/O_2_(g)/H_2_O(g)/N_2_(g) mixtures at [O_3_(g)] ∼600 ppmv, irradiated with 266 nm laser beams as functions of laser energy (in mJ pulse^−1^). Background (before pulsing at 266 nm) products signals were subtracted.

Figure 3 shows reactant decay and product enhancements upon irradiation in similar experiments as functions of 266 nm pulse energy. In Fig. 3, laser energies (*x*-axis) at 1, 5, 10, 20, 30, and 40 mJ pulse^−1^ correspond to [·OH(g)]_0_≈2, 9, 18, 33, 46, and 57 ppmv, respectively. The actual [·OH] colliding to the microjets are expected to be smaller than these estimated values.^34,35)^ It is apparent that the full extent of irradiation effects is already reached at the lowest pulse energies. Further increases in pulse energy only achieve the partial degradation of the *n*=1 and 3 [SP-B_1–25_+4 H+*n* O]^4+^ products. [SP-B_1–25_+4 H+3 O]^4+^ in particular disappears above ∼20 mJ pulse, possibly *via* photo-degradation or secondary reactions. The effect of laser energy on product formation (Fig. 3) is markedly different from that observed upon variations of [O_3_(g)] (Fig. 2): SP-B_1–25_ decays bi-exponentially as a function of laser energy, while some products reach steady state at the lowest fluences, such as [SP-B_1–25_+4 H+2 O]^4+^, or are degraded, such as [SP-B_1–25_+4 H+O]^4+^ and [SP-B_1–25_+4 H+3 O]^4+^, at larger fluences.

Our results are consistent with the rapid incorporation of up to 5 O-atoms to SP-B_1–25_ by both O_3_ and ·OH at the air–water interface. Since (i) the reported rate constants for the reactions of cysteine (*k*_Cys+ozone_>7.0×10^6^ M^−1^ s^−1^) and tryptophan (*k*_Trp+ozone_=7.0×10^6^ M^−1^ s^−1^) with O_3_ in bulk water are >10^2^ times larger than for the rest of the amino acids (except for methionine),^48)^ (ii) the corresponding reactions with ·OH (*k*_Trp+·OH_=1.3×10^10^ M^−1^ s^−1^), (k_Cys+·OH_=3.5×10^10^ M^−1^ s^−1^) are diffusionally controlled, (iii) both cysteines Cys_8_ and Cys_11_, and tryptophan Trp_9_, are embedded in the hydrophobic section of SP-B_1–25_ that resides at the topmost layers of air–water interface,^3,54)^ we infer that these are the three amino acid residues attacked by both O_3_ and ·OH. More specifically, we envision that in the initial stages of the oxidation process O_3_ will transfer one O-atom to the sulfhydryl R-S-H group of Cys to produce the corresponding sulfenic acid R-S-OH,^55)^ whereas O_3_ also adds to the pyrrole ring of Trp to produce the corresponding primary POZ and secondary SOZ Trp-O_3_ ozonides (Scheme S1). We also consider that the S-containing methionine Met_21_ residue,^56)^ by being situated in the hydrophilic C-terminal side buried in water (as shown by MD simulations by Goddard and coworkers),^3)^ is less likely to be oxidized by both O_3_ and ·OH than the Cys residues closer to the air–water interface. The fact that both O_3_ and ·OH preferably reside at the air–water interface rather than in bulk^44–46)^ makes it less likely that they will diffuse towards the Met_21_ residue located in bulk.

We also tested whether the mechanism of Trp ozonation at the air–water interface follows the same course as in bulk water in our experimental setup by exposing free L-tryptophan (aq) to O_3_(g)/O_2_(g)/H_2_O(g)/N_2_(g) mixtures in the absence/presence of 266 nm pulses (Fig. S5). We found that the interfacial ozonation of anionic L-tryptophan (*m*/*z*^−^=203) produces species that incorporate one to three O-atoms, which are detected at *m*/*z*^−^=219 (+1O), 235 (+2O) and 251 (+3O), along with a signal at *m*/*z*^−^=207. These species match the masses of the reported products of Trp ozonation^3,57)^ and hydroxylation^58,59)^ in bulk water. See Scheme S1 for assignments. We note, however, that the formation of kynurenine (Kyn) from the oxidation of Trp_9_ in SP-B_1–25_, which would have led to a peak at *m*/*z*^+^=734=[2928+48 (+3O)−44 (−CO_2_)+4 (+4H)]/4, is absent from our mass spectra (Fig. 1B). Thus, from the fact that we observe the incorporation of up to 5 O-atoms into SP-B_1–25_, we conclude that Trp_9_ accepts 3 O-atoms, and Cys_8_ and Cys_11_ one O-atom each during the ozonation and hydroxylation of SP-B_1–25_ at the air–water interface under present conditions. Note that H-abstraction from Trp by ·OH in the presence of O_2_ would have led to the formation of a peroxyl radical, *m*/*z*^−^=203−1 (−H)+32 (+O_2_)=234, and possibly to an alcohol *m*/*z*^−^=234−16+1=219, and carbonyl *m*/*z*^−^=234−16−1=217, from the disproportionation of the peroxyl radical, as in the case of alkyl and aromatic carboxylic acids.^31,32,34,35)^ Their conspicuous absence proves that under present conditions all oxidation processes, both those initiated by ozonation and hydroxylation, are initiated by O-atom transfers or ·OH additions to the S-center of the Cys and the pyrrole ring of Trp, rather than by H-atom abstraction from the myriad C–H bonds available in SP-B_1–25_. The exceptional reactivity of S-atoms in biomolecules for ⋅OH-addition is consistent with our GSH+·OH and GSSG+·OH studies at the air–water interface,^36)^ suggesting that interface-specific phenomena are general and stem from the peculiar nature of interfacial water as a reaction medium.^26,60–62)^ We also note that these highly selective oxidations imply “molecular recognition” processes, possibly mediated by water networks.^63,64)^ Thus, our results suggest that these hitherto unknown interface-specific radical recognition processes may play central roles in lung surface chemistry.

The occurrence of up to 5 O-atom transfers to a single SP-B_1–25_ unit implies that oxidants are always in excess over reactive centers at the interface. Thus, the initial attack on any one of them is followed by additional, successive O-atom transfers until all such centers reach their limiting degree of oxidation during contact times. However, since we have shown that GSH and free cysteine can add up to 3 O-atoms under similar conditions,^36,55)^ it appears that first-generation sulfenic acids S(O)–H are not further oxidized in SP-B_1–25._ This is because they may rapidly form S–S bridges or, due to conformational changes, they become buried in the hydrophilic segment of the polypeptide.^65)^ In this context, we note that the fate of sulfenic acid could be determined by the microenvironment.^65,66)^ For example, a S–OH can persist for several hours in human serum albumin, in which 34 of 35 cysteine-resides can create disulfide-bonds thereby minimizing the number of available reduced S-atoms.^67)^ Another study revealed that the hypervalent S-atoms of sulfenic acids can form covalent complexes with the N-atoms of neighboring histidine residues, a phenomenon that prevents the over-oxidation of cysteine sulfenic acid.^68)^ Therefore, it is conceivable that neighboring Trp_9_’s indole-N-atoms may stabilize the Cys_8_-OH and Cys_11_-OH in a similar way. An additional effect is that since sulfinic S(O_2_)–H (p*K*_a_≈2) and sulfonic acids S(O_2_)–OH (p*K*_a_<1) are much more acidic than S(O)–H (p*K*_a_≈7.6),^55)^ they would deprotonate at pH ∼6 thereby decreasing the net charge of [SP-B_1–25_+*m* H+O]*^m^*^+^ from *m*→(*m*−1) and shifting the mass signals. However, the fact that we did not observe such behavior (Fig. S3) led us to exclude the oxidation of Cys-sulfenic acid residues.

The mechanism of SP-B_1–25_ oxidations shown here is generally consistent with previous studies.^3,6,47)^ However, the previous report using the Fenton reaction for oxidizing SP-B_1–25_ by ·OH in bulk water showed O*_n_*-products up to *n*=10, which is in contrast with our observations (*n*=1–5).^3)^ We ascribe this to the following reasons: 1) differences between bulk *vs.* interfacial mechanism, 2) the very different time scales between the two systems, and 3) the Fenton reaction may largely produce oxo-ferryls rather than ·OH. First, as observed in GSH/GSSH+·OH experiments,^36)^ the difference indeed comes from where the ·OH-reaction occurs, that is, in water *vs.* at the air–water interface. We recently reported that ·OH-reaction of amphiphilic species are remarkably different in bulk water *vs.* at the air–water interface.^32,34,35)^ Second, in the previous report the measurement was performed >12 h after the Fenton reaction starts,^3)^ while in the present study the time scale is <10 μs. Third, as mentioned above, the Fenton reaction may produce oxo-ferryl species in >90% yields in bulk water at pH 6–7,^28)^ and even more so at the air–water interface.^26)^ In the present work, ·OH is generated by well-established gas-phase photo-reactions.^31)^

Hydroxylation, by increasing the hydrophilicity of the N-terminus, is generally expected to degrade the tensioactive properties of SP-B.^6,47,69)^ The formation of intra/intermolecular S–S bonding *via* sulfenic R-S-OH groups, that likely occurs in a longer time scale, could also disturb SP-B function as surfactant.^5,6,13)^ Since inhalation of PM and O_3_ evidently induces reactive oxygen species (ROS) in ELF of our lungs,^20)^ the present results are directly linked to adverse health effects of air pollutants impairing the role of SP-B as the surfactant.

## CONCLUSION

We report a mass spectrometric study on how aqueous SP-B_1–25_ is oxidized by O_3_ and ·OH at the air–water interface. By using a novel method that combines online pneumatic ionization mass spectrometry with pulse laser flash photolysis,^31)^ we were able to detect the intermediates/products generated in the initial stages of the oxidation of SP-B_1–25_ by ·OH on water surfaces. Our results suggest that two Cys-residues and a Trp-residue in hydrophobic N-terminal side are the major targets for both O_3_ and ·OH, rather than H-atom abstraction from the multiple C–H/N–H bonds available in SP-B_1–25_. We infer that this remarkable interface-specific radical recognition process is what determines the observed chemistry. These chemical transformations increase the polarity of the SP-B_1–25_ hydrophobic section, promote the formation of disulfide links therein and, therefore, are deemed to impair its role as the surfactant that prevents lung collapse upon expiration.

## SUPPORTING INFORMATION AVAILABLE

Additional data and experimental details. This material is available free of charge *via* the Internet.

## Notes

Mass Spectrom (Tokyo) 2018; 7(2): S0077
