# Supplementary material for: OH-Radical Oxidation of Lung Surfactant Protein B on Aqueous Surfaces
Source: Mass Spectrom (Tokyo). 2018 Nov 21;7(2):S0077. doi: 10.5702/massspectrometry.S0077 (PMC6245955; doi:10.5702/massspectrometry.S0077)
Supplement: Supplementary Information [file massspectrometry-7-2-S0077-s001.pdf]

Supporting Information:

OH-radical Oxidation of Lung Surfactant Protein B on Aqueous Surfaces

Shinichi Enami*[,1] and Agustín J. Colussi[b,2]

[1]*National Institute for Environmental Studies, 16-2 Onogawa, Tsukuba 305-8506, Japan*

[2]*Linde Center for Global Environmental Science, California Institute of Technology, California 91125, U.S.A*

**Author to whom correspondence should be addressed:*

S.E. enami.shinichi@nies.go.jp, phone: +81-29-850-2770

SI Text

Ozone is produced from ultrapure O_2 (purity > 99.995 %) flowing at 1.0 standard liters per minute (SLM) through a commercial discharge ozonizer (KSQ-050, Kotohira). O_3(g) concentration is determined by a UV-Vis absorption spectrophotometry at 250 nm and 300 nm,[1] before flowing to the reaction chamber (Fig. S1). Throughout, the reported [O_3(g)] values correspond to the concentrations actually diluted by the drying gas in the reaction chamber, which are 10 or 11 times smaller than those determined upstream UV absorbances. H_2O(g) is constantly added by sparging milli-Q water with N_2(g) carrier gas at 0.2 L/min, whose flow is measured by a mass flow controller (Horiba, STEC).

The 266 nm radiation emitted by a Nd^{3+}:YAG laser setup (LOTIS TII, LS-2131M-10) through the harmonic generator assembly HG-TF (pulse duration 8 $\pm$ 1 ns, 266 nm beam diameter 10.0 $\pm$ 1.0 mm, beam divergence ≤ 1.5 mrad, 10 Hz) is utilized to produce ·OH(g) *in situ* (Fig. S1). [·OH(g)] were estimated from experimental parameters and reported kinetic data (see [·OH(g)] estimates). The 266 nm laser beam energies (mJ pulse^{-1}) are measured by a power meter (OPHIR, NOVA II, sensor:3A-P-V1-ROHS). The beam energy at 266 nm is manually controlled from 0 to 40 mJ pulse^{-1}. The laser beam is introduced into the spraying chamber via several quartz prisms (synthetic fused silica, refractive index n_d = 1.458) on kinematic prism holders (SIGMAKOKI Co., LTD., Japan) and finely aligned with a He-Ne laser (Melles Griot, 05-LHP-111, 632.8 nm CW) beam, which becomes visible as it is scattered upon hitting the liquid jet.

Typical conditions in the present experiments were: drying gas flow rate: 12 or 13 L min^{-1}; drying gas temperature: 340 ^oC; inlet voltage: - 3.5 kV relative to ground; fragmentor voltage value: 60 or 80 V. SP-B_{1-25} (purity > 90 %) was purchased from

Biomer Technology (CA, USA) and stored at 253 K. Fresh SP-B_{1-25}(aq) solutions were used within a couple of days. L-tryptophan (> 99 %) was purchased from Nacalai Tesque (Kyoto). All solutions were prepared in purified water (Resistivity ≥18.2 MΩ cm at 298 K) from a Millipore Milli-Q water purification system. The pH of the injected solutions was measured before measurement with a calibrated pH-meter, Horiba LAQUA F-74. SP-B_{1-25} solutions were slightly acidic, e.g., pH of 43 μM SP-B_{1-25} solution was 5.8.

[·OH(g)] estimates

The concentration of ·OH hitting the microjet can be derived from the O_3(g) absorption cross sections, laser fluence, and reported gas-phase kinetic parameters. The dissociation of O_3(g) by 266 nm laser photons into O(^{1}D), followed by the reaction of O(^{1}D) with H_2O(g) promptly generates ·OH(g), in competition with its deactivation by N_2(g) and O_2(g) into O(^{3}P).[2] These gas-phase kinetics are well established. We could estimate that under experimental conditions 0.1 ~ 10 % O_3(g) is converted into ·OH(g). Since number of photons is always larger than number of O_3(g) molecules under present conditions, we estimate the initial O(^{1}D) concentrations from 266 nm photolysis from Beer's law:

$$\ln(N_0/N) = I_0\ \sigma\ \Phi_{dis} \quad \text{(E 1)}$$

$$N = N_0\ exp(-I_0\ \sigma\ \Phi_{dis}) \quad \text{(E 2)}$$

where σ is the absorption cross section, Φ_{dis} is the dissociation quantum yield, I_0 is the laser fluence in number of photons per unit area, N_0 is the number of molecules before laser irradiation, and N is the number of molecules after laser irradiation.[3] We derive N/N_0 ~ 0.5, meaning $[O(^1D)]_0 \approx 0.5 \times [O_3(g)]$ at the highest 266 nm laser pulse energy ~

40 mJ pulse^{-1} under the present condition. $O(^1D)$ reacts with excess $H_2O(g)$ ($[H_2O(g)] \sim 7.6 \times 10^{17}$ molecule cm^{-3}) to form ·OH radical within ~6 ns (from $k_1 = 2.2 \times 10^{-10}$ cm^3 molecule^{-1} s^{-1}), reaction R 1[1,4];

$$O(^1D) + H_2O \rightarrow 2\,\cdot OH \qquad (1)$$

or, is competitively deactivated by N_2 and O_2, reactions R 2a and R 2b

$$O(^1D) + N_2 \rightarrow O(^3P) + N_2 \qquad (2a)$$

$$O(^1D) + O_2 \rightarrow O(^3P) + O_2 \qquad (2b)$$

where $k_{2a} = 2.6 \times 10^{-11}$, $k_{2b} = 4.0 \times 10^{-11}$ cm^3 molecule^{-1} s^{-1}, respectively.[1,4] Note that the pressure in the reaction chamber is 1 atm. $O(^3P)$ is largely consumed by O_2 to regenerate O_3 ($\tau \sim 36$ μs) under present condition. We could estimate that ~ 20 % $O(^1D)$ is converted into ·OH radicals. $[\cdot OH(g)]_0$ was varied from a few tens of ppbv to 100 ppmv under present conditions. Note that $[\cdot OH(g)]$ values are upper limits to $[\cdot OH]$ on the surface of microjets. See our previous reports for details.[5,6]

The secondary chemistry induced by the reaction of ·OH with O_3 ($k = 7.3 \times 10^{-14}$ cm^3 molecule^{-1} s^{-1}) is negligible since the lifetime of ·OH by this reaction is > 500 μs (cf. < 10 μs of lifetime of microjets). It should be emphasized that the reaction of $O(^1D)$ with SP-$B_{1\text{-}25}$(aq) is negligible. Even if we assume an extremely large rate constant for such process, e.g., $k = 6 \times 10^{-10}$ cm^3 molecule^{-1} s^{-1},[7] this $O(^1D)$ reaction channel at [SP-$B_{1\text{-}25}$(aq)] = 30 μM = 6.0×10^{16} molecules cm^{-3}, is still > 60 times slower than the gas phase reactions R1, 2a and 2b. Note that even if $O(^1D)$ could survive via reactions R1, 2a and 2b in the gas-phase, $O(^1D)$ will be quantitatively converted into 2 ·OH radicals at the air-water interface where water is in exceedingly large excess over sub-mM SP-$B_{1\text{-}25}$. Thus, the reactions we observe are necessarily driven by ·OH at the air-water interface.

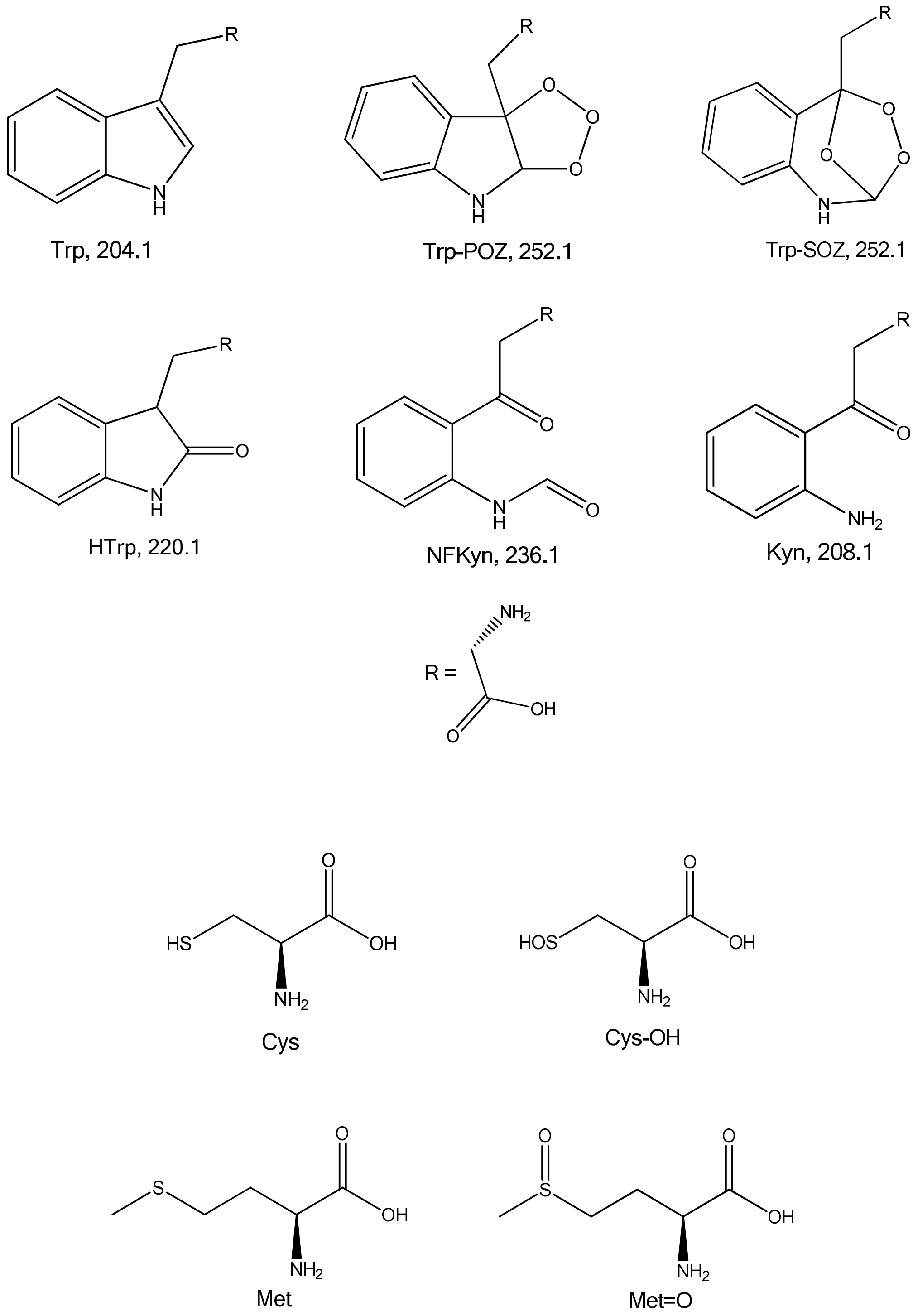
Trp, 204.1
Trp-POZ, 252.1
Trp-SOZ, 252.1
HTrp, 220.1
NFKyn, 236.1
Kyn, 208.1
R =
Cys
Cys-OH
Met
Met=O

SCHEME S1

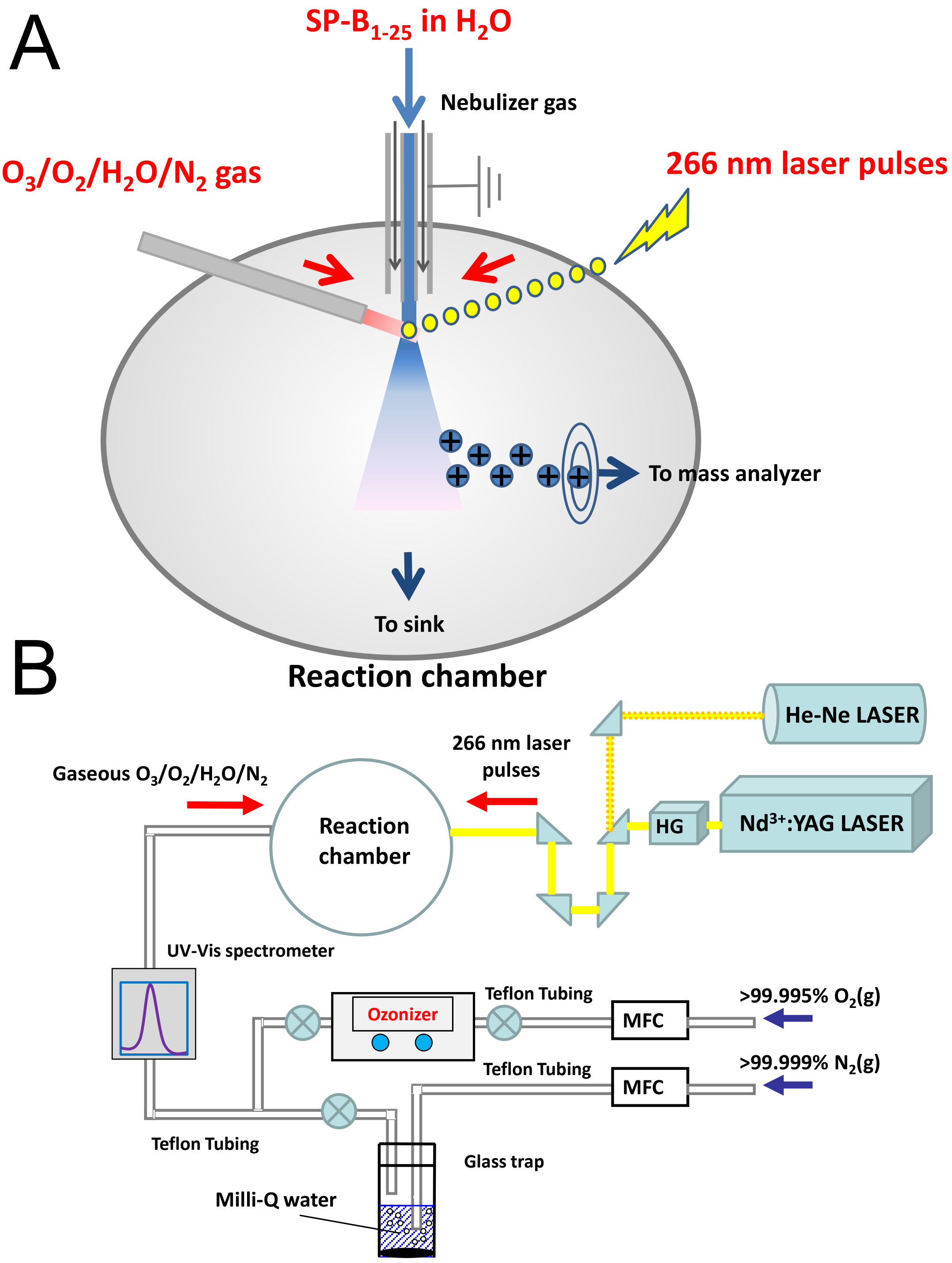

Figure S1 - Schematic diagram of setup used to study laser-induced OH-radical reactions at the air-water interface. HG and MFC mean harmonic generator and mass flow controller, respectively.

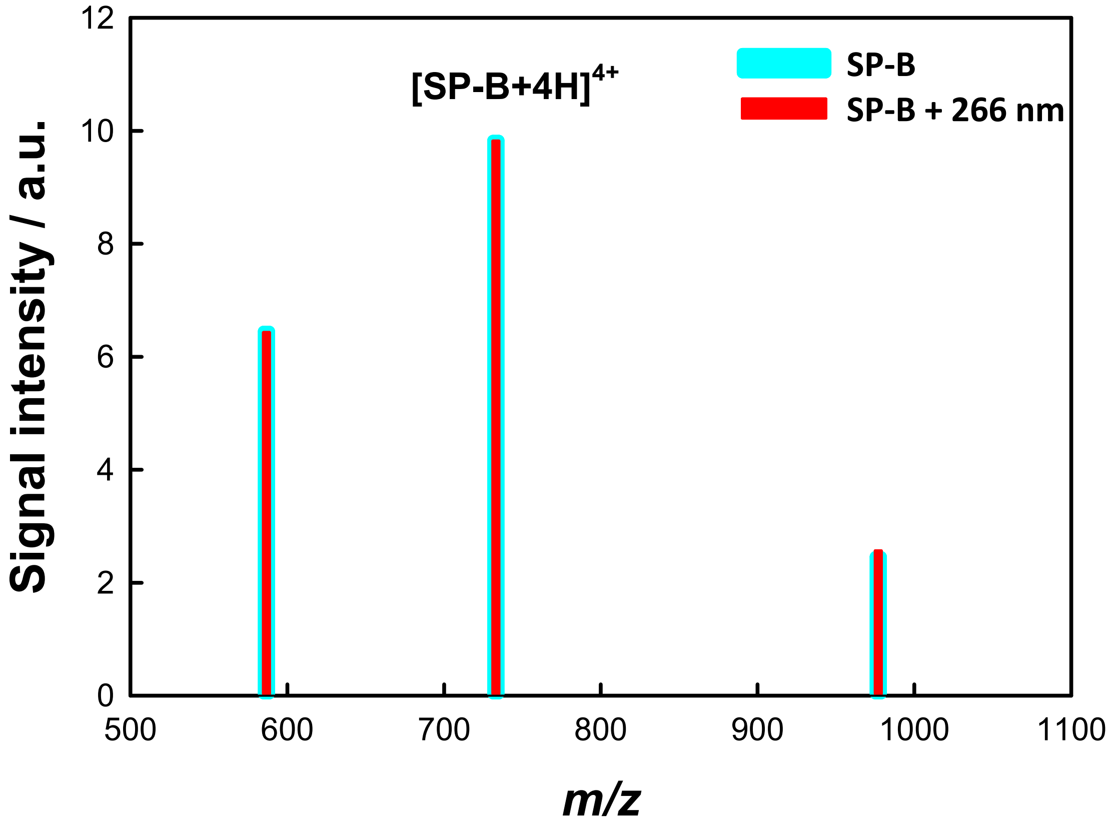

Figure S2 Positive ion mass spectra of 43 μM SP-$B_{1\text{-}25}$ microjets at *m/z* = 587, 733 and 977 (measured by selective ion mode) in the absence (cyan)/presence (red) of 266 nm laser beam irradiation at 40 mJ pulse^{-1} (maximum power) under $H_2O/O_2/N_2$ atmosphere.

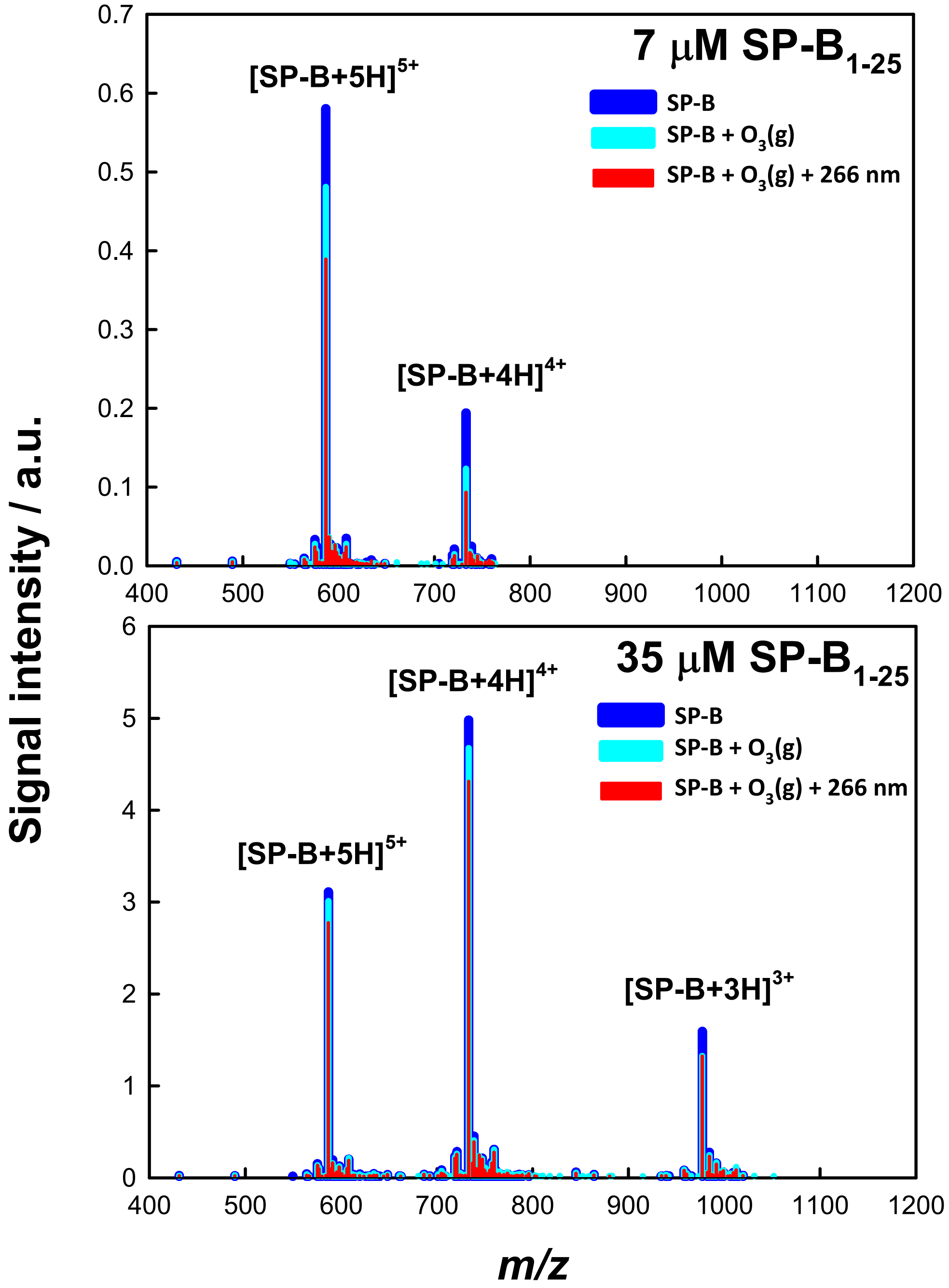

Figure S3 Positive ion mass spectra of aqueous 7 μM (upper panel) or 35 μM (lower panel) SP-B_{1-25} microjets without (blue) or exposed to 230 or 135 ppmv O_3(g), respectively, in O_2(g)/H_2O(g)/N_2(g) mixtures at 1 atm. Cyan: laser off. Red: under 40 mJ, ~ 8 ns pulses (at 10 Hz) of 266 nm radiation. 1 ppmv = 2.46 × 10^{13} molecules cm^{-3}.

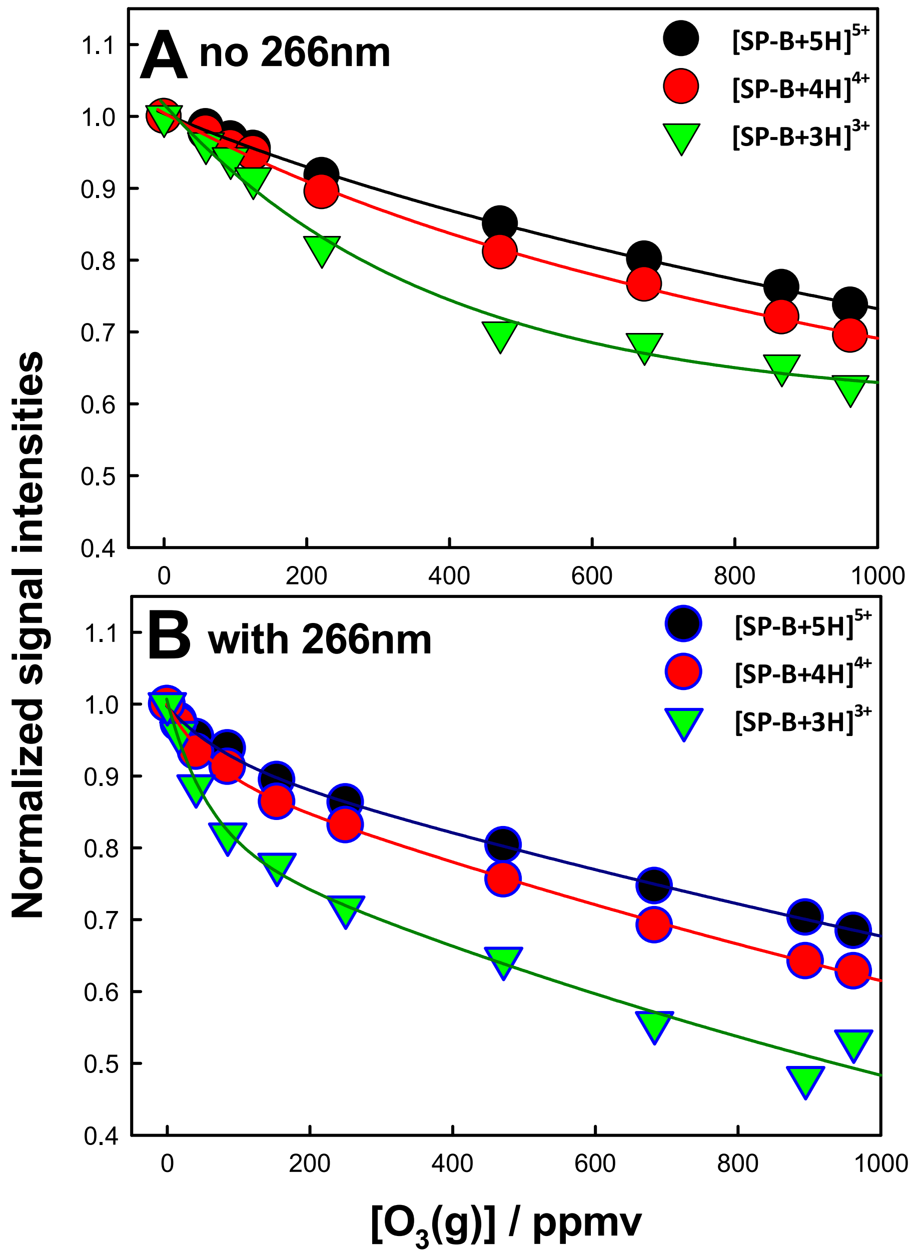

Figure S4 – Normalized mass spectral reactant signal intensities from aqueous 43 μM SP-B_{1-25} microjets exposed to O_3(g)/O_2(g)/H_2O(g)/N_2(g) mixtures without (A) or with (B) irradiation by 266 nm laser beams (40 mJ pulse^{-1}) as a function of the O_3(g) mixing ratio; 1 ppmv = 2.46 × 10^{13} molecules cm^{-3}.

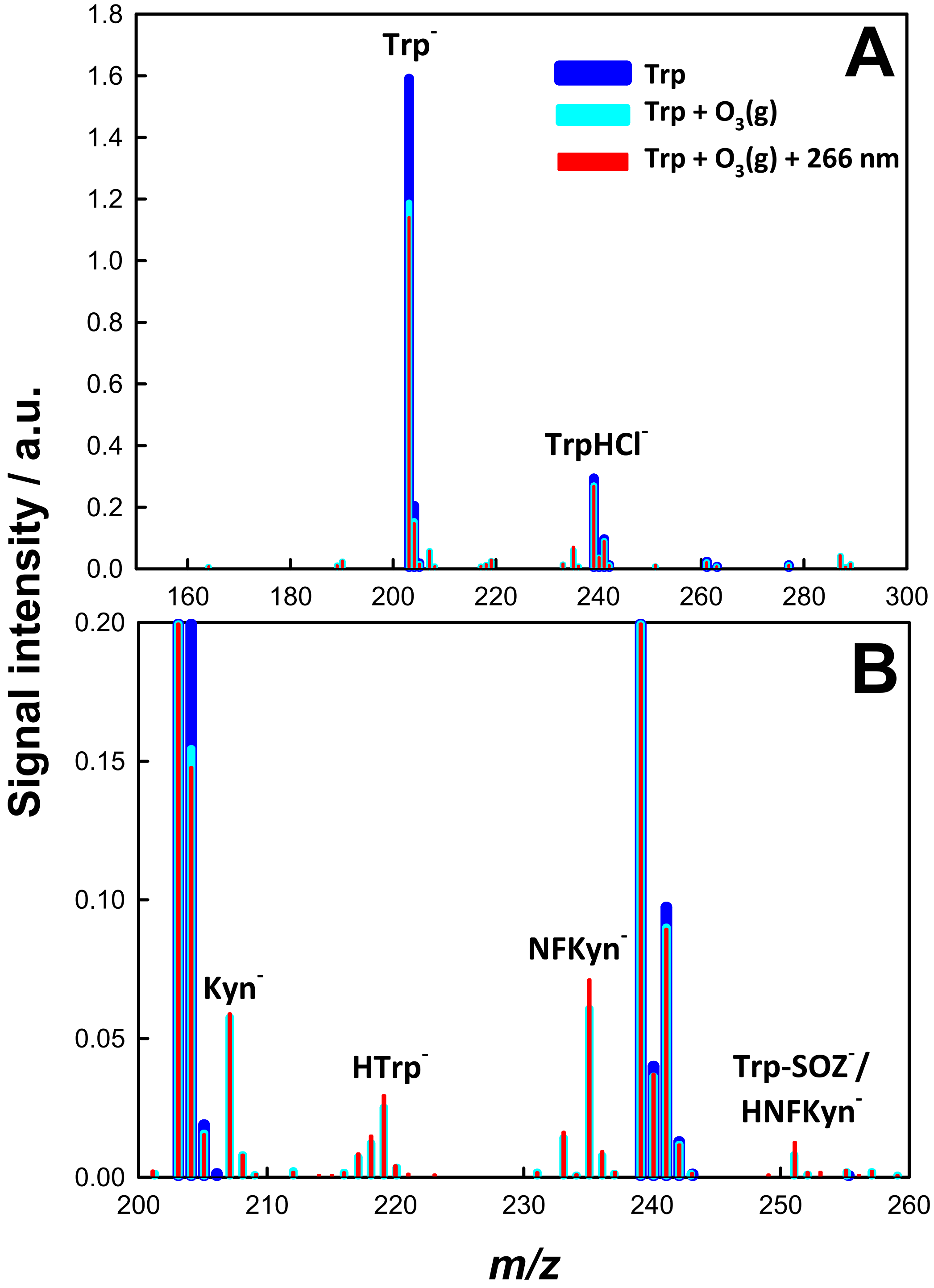

Figure S5 – A) Negative ion mass spectra of 2 mM L-tryptophan (pH 8.7) microjets exposed to 83 ppmv O_3(g)/O_2(g)/H_2O(g)/N_2(g) mixtures at 1 atm and 298 K. Cyan: laser off. Red: under 40 mJ, ~ 8 ns pulses (at 10 Hz) of 266 nm radiation ($[\cdot OH]_0$ ~ 8 ppmv). 1 ppmv = 2.46×10^{13} molecules cm^{-3}. B) Zooming-up spectrum of oxidation products in the 200 - 260 Da range.

SI REFERENCES

1 Sander, S. P. *et al. Chemical Kinetics and Photochemical Data for Use in Stratospheric Modeling Supplement to Evaluation 12: Update of Key Reactions Evaluation Number 13.* (2000).

2 Grebenshchikov, S. Y., Qu, Z. W., Zhu, H. & Schinke, R. New theoretical investigations of the photodissociation of ozone in the Hartley, Huggins, Chappuis, and Wulf bands. *Phys. Chem. Chem. Phys.* **9**, 2044-2064, doi:10.1039/b701020f (2007).

3 Lin, J. J., Chen, A. F. & Lee, Y. T. UV Photolysis of ClOOCl and the Ozone Hole. *Chemistry-an Asian J.* **6**, 1664-1678, doi:10.1002/asia.201100151 (2011).

4 National Institute of Standards and Technology Standard Reference Database Number 69. (2009).

5 Enami, S. & Sakamoto, Y. OH-Radical Oxidation of Surface-Active cis-Pinonic Acid at the Air–Water Interface. *J. Phys. Chem. A* **120**, 3578-3587, doi:10.1021/acs.jpca.6b01261 (2016).

6 Enami, S., Hoffmann, M. R. & Colussi, A. J. Extensive H-atom abstraction from benzoate by OH-radicals at the air-water interface. *Phys. Chem. Chem. Phys.* **18**, 31505-31512, doi:10.1039/C6CP06652F (2016).

7 Dillon, T. J., Horowitz, A. & Crowley, J. N. The atmospheric chemistry of sulphuryl fluoride, SO2F2. *Atmos. Chem. Phys.* **8**, 1547-1557 (2008).
